# Editorial for *Special Issue* “Natural Alternatives against Bacterial Foodborne Pathogens”

**DOI:** 10.3390/microorganisms8050762

**Published:** 2020-05-20

**Authors:** Adolfo J. Martinez-Rodriguez, Jose Manuel Silvan

**Affiliations:** Microbiology and Food Biocatalysis Group, Department of Biotechnology and Food Microbiology, Institute of Food Science Research (CIAL), CSIC-UAM-C/Nicolás Cabrera, 9. Cantoblanco Campus, Autonoma University of Madrid, 28049 Madrid, Spain

In recent years, increased resistance to antibiotics and disinfectants from foodborne bacterial pathogens has become a relevant consumer health issue and a growing concern for food safety authorities. In this situation, and with an apparent stagnation in the development of broad-spectrum antibiotics, research into new antibacterial agents and strategies for the control of foodborne pathogens that have good acceptability, low toxicity levels, and high sustainability is greatly demanded at present.

This *Special Issue* on “Natural Alternatives against Bacterial Foodborne Pathogens” aims to contribute to the visibility of some of these new antibacterial agents and contains eight research articles and one review, presenting different strategies potentially applicable in the control of various foodborne pathogens.

The antibacterial properties of extra virgin olive oil against different foodborne pathogens and their relationship with phenolic composition of the extract are described by Nazzaro et al. [1]. This study may contribute to the design of optimal mixtures of polyphenols with improved antibacterial efficacy. The paper by Silvan et al. [2] reports that plum extract powders gained after freeze-, vacuum- and spray-drying have promising antibacterial, antioxidant, and anti-inflammatory properties, demonstrating that the drying method selected can be an effective tool for modulating the composition, physical, and bioactive properties of plum extracts powders. The antimicrobial effect of essential oils obtained from cinnamon, marjoram, and thyme on single and dual biofilms of *Escherichia coli*, *Listeria monocytogenes*, *Pseudomonas putida*, and *Staphylococcus aureus* is described by Kerekes et al. [3]. These studies are the starting point for new approaches, such as encapsulation of essential oils, that could potentially reduce its organoleptic impact and increase antibacterial activity. Following a different strategy, Speranza et al. [4] proposes to exploit the in vivo metabolism of two probiotic strains (*Bifidobacterium longum* subsp. *infantis* and *Lactobacillus reuteri*) with the capacity to adhere on different surfaces (i.e., packaging materials, ceramic, plastic, paper, polymers, etc) forming a biofilm able to control the growth of pathogenic and food spoilage bacteria. This could be useful as a new biocontrol solution for different industrial applications. The probiotic functionality of a *Bacillus subtilis* strain protecting probiotic lactic acid bacteria during their exposure to unfavorable environmental conditions, such as desiccation and acid stresses, is described by Kimelman and Shemesh [5]. In addition to this protective capability, *B. subtilis* strains have demonstrated a potent antimicrobial activity against pathogenic *S. aureus*. Luis et al. [6] report the development of hydrophobic zein-based functional films incorporating licorice essential oil as new alternative materials for food packaging. These new films are biodegradable and possess antioxidant and antibacterial properties against different foodborne pathogens, making them potential alternatives to the conventional plastics used in food packaging solutions, reducing environmental pollution and increasing the shelf-life of foods. Zhang et al. [7] present the antibacterial activity of different spice extracts against several antibiotic resistant strains of foodborne pathogens. They conclude that some extracts with relevant antibacterial and antioxidant activity could have potential for use as both antibiotic alternatives in animal feeding and as a natural food preservative in the food industry. Kaewkod et al. [8] report the study of different biological properties of Kombucha tea from various kinds of tea leaves including green, oolong, and black tea. They observe that the extent of the antibacterial effect against several foodborne pathogens was related with the amount of organic acids in the beverage, indicating the great potential health benefits of Kombucha tea. Finally, Zorraquin-Peña et al. [9] present a detailed review on the main applications of silver nanoparticles as antibacterial agents for food control, as well as the current legislation concerning these materials. They also summarize the current knowledge about the impact of dietary exposure to silver nanoparticles in human health, with special emphasis on the changes that nanoparticles undergo after passing through the gastrointestinal tract and how they alter the oral and gut microbiota.
